# Do Centenarians Die Healthier than Younger Elders? A Comparative Epidemiological Study in Spain

**DOI:** 10.3390/jcm9051563

**Published:** 2020-05-21

**Authors:** Mercedes Clerencia-Sierra, Ignatios Ioakeim-Skoufa, Beatriz Poblador-Plou, Francisca González-Rubio, Mercedes Aza-Pascual-Salcedo, Mónica Machón, Antonio Gimeno-Miguel, Alexandra Prados-Torres

**Affiliations:** 1 Aragon Health Service (SALUD), EpiChron Research Group, 50009 Zaragoza, Spain; mclerencia@salud.aragon.es (M.C.-S.); fgonzalez@salud.aragon.es (F.G.-R.); maza@salud.aragon.es (M.A.-P.-S.); 2 Red de Investigación en Servicios de Salud en Enfermedades Crónicas (REDISSEC), 28222 Madrid, Spain; bpoblador.iacs@aragon.es (B.P.-P.); monica.machonsobrado@osakidetza.eus (M.M.); sprados.iacs@aragon.es (A.P.-T.); 3 EpiChron Research Group, IIS Aragón, 50009 Zaragoza, Spain; ignacio.ioakim@hotmail.es; 4 EpiChron Research Group, Aragon Health Sciences Institute (IACS), IIS Aragón, Miguel Servet University Hospital, 50009 Zaragoza, Spain; 5 Instituto de Investigación Sanitaria Biodonostia, Grupo de Atención Primaria, 20014 San Sebastián, Spain; 6 Instituto de Investigación en Servicios de Salud Kronikgune, 48902 Barakaldo, Spain

**Keywords:** aged 80 and over, delivery of healthcare, electronic health records, multimorbidity, multiple chronic conditions, polypharmacy, real-world data

## Abstract

This study aims to describe the clinical course, drug use, and health services use characteristics during the last year of life of elders who die being centenarians and to identify key aspects differentiating them from elders who die at an earlier age, with a particular focus on sex differences. We conducted an observational, population-based study in the EpiChron Cohort (Aragón, Spain). The population was stratified by sex and into three age sub-populations (80–89, 90–99, and ≥100 years), and their characteristics were described and compared. Multimorbidity was the rule in our elders, affecting up to 3 in 4 centenarians and 9 in 10 octogenarians and nonagenarians. Polypharmacy was also observed in half of the centenarian population and in most of the younger elders. Risk factors for cardiovascular disease (i.e., hypertension, dyslipidaemia, diabetes), cerebrovascular disease and dementia were amongst the most common chronic conditions in all age groups, whereas the gastroprotective drugs and antithrombotic agents were the most dispensed drugs. Centenarians presented in general lower morbidity and treatment burden and lower use of both primary and hospital healthcare services than octogenarians and nonagenarians, suggesting a better health status. Sex-differences in their clinical characteristics were more striking in octogenarians and tended to decrease with age.

## 1. Introduction

The extraordinary rise in life expectancy in developed countries during the past decades has resulted in an exponential increase in the centenarian population. The United Nations estimated that there would be more than 25 million centenarians in 2100 [1]. This underscores the importance of studying this age group to characterize their biological, genetic, social, clinical, and epidemiological profile. On the one hand, to shed some light on the mechanisms and factors involved in the ability of an individual to extend life span; on the other hand, to obtain useful information for the design of person-centered care models adapted to their characteristics to decrease the medical and economic impact associated with ageing.

Centenarians have been studied from different perspectives; however, several gaps in knowledge remain unfilled. A density analysis (total number of life durations per birth date) in deceased Olympic athletes and deceased supercentenarians (individuals aged 110 years or older) revealed similar life-span trends toward increased life duration, as well as similar mortality pressures that increase with age [2]. Several studies have characterized the burden of diseases (multimorbidity) and drugs (polypharmacy) and the use of health services in centenarians with contradictory results [3,4,5,6,7,8,9,10,11,12]. Two central hypotheses have been proposed regarding morbidity burden and disability in the oldest old population. The compression of morbidity hypothesis suggests that morbidity and disability could be compressed into a shorter duration of time before death if the onset of chronic illness could be postponed [13]. The expansion of morbidity hypothesis predicts increased morbidity and an increasing number of years spent in poor health [14,15]. Assessing trends in compression or expansion of morbidity would provide useful epidemiological information with clinical implications [16].

In our opinion, comparing the clinical-epidemiological characteristics of centenarians with those elders who are not able to reach age 100 may help to identify potential underlying factors of shorter longevity. On the other hand, addressing the sex and gender perspective in elders is also necessary to understand the role of sex in the process of ageing, and to identify potential gender-related inequalities in the health [17] and healthcare [18] of older adults. In this context, the use of real-world data routinely generated in daily practice during the process of care, such as electronic health records (EHRs), may represent an excellent opportunity to study centenarians through the conduct of large-scale, population-based studies.

This study aims to describe the demographic, clinical, drug use and healthcare use characteristics during the last year of life of elders who die being centenarians and to identify clinical key aspects differentiating them from elders who die at an early age being nonagenarians or octogenarians, with a specific focus on sex differences.

## 2. Materials and Methods

### 2.1. Design and Study Population

We conducted an observational, population-based study using real-world data (i.e., the information contained in EHRs and clinical-administrative databases) in the EpiChron Cohort [19]. This cohort links, at the patient level, demographic, clinical, drug dispensation, and health services use information of the public health system users of the Spanish region of Aragón. The reference population of the cohort was 1,253,292 individuals on 1 January 2011, representing approximately 98% of total inhabitants in the region. The anonymized data used in the study were obtained from the user database, primary, specialist, hospital and emergency care EHRs, and pharmacy billing database. A more detailed description of the cohort profile and of the data sources was published elsewhere [19].

We selected for this study all the subjects from the cohort who had died at the age of 80 years or older from 1 January 2011 to 31 December 2015. The study population was stratified by sex and into three age sub-populations: 80–89 years, 90–99 years, and ≥100 years. Since we aimed to study the health profile during the last stage of life of older adults capable of becoming centenarians and compare with that of elders who were not able to reach age 100, we excluded from the study the elders who remained alive at the end of the study period. We only included in the study patients with at least one contact with the health system registered in their EHRs.

The information analyzed for each participant included: sex, age, area of residence (urban or rural), deprivation index of the area calculated according to 26 socio-economic indicators and arranged from less (Quartile 1, Q1) to most (Quartile 4, Q4) deprived [20], all chronic disease diagnoses registered in primary and/or hospital EHRs, all chronic medications dispensed, use of potentially inappropriate medications according to the updated Beers criteria [21], and health services use rates. All the information analyzed corresponded to the last 365 days of follow-up of each individual. Diagnoses were grouped in Expanded Diagnostic Clusters (EDCs) based on the Johns Hopkins Adjusted Clinical Groups (ACG^®^) System (Version 11.0, The Johns Hopkins University, Baltimore, MD, USA). This classification software is useful in studies on multimorbidity to count diseases when diagnoses from different sources and codification systems are used (e.g., International Classification of Primary Care-ICPC-codes from primary care and International Classification of Diseases-ICD-codes from hospitals). We defined multimorbidity as the co-occurrence of more than one disease from those of the list of 114 EDCs defined as chronic by Salisbury et al. [22]. We defined chronic medications as those with three or more dispensations over the 365-days follow-up period, using the Anatomical Therapeutic Chemical (ATC) classification system code at the third level. We calculated the anticholinergic drug scale score [23] and the anticholinergic cognitive burden [24] for each individual based on their medical prescriptions.

### 2.2. Statistical Analysis

We performed a descriptive analysis of demographic, clinical, health services use, and drug use characteristics of centenarians by sex and compared them with those of men and women aged 80–89 and 90–99 years. The results were calculated as means and/or frequencies accompanied by their 95% confidence intervals. For the analysis of the differences among the three age groups, we used the Kruskal–Wallis test to compare means and the Pearson’s chi-squared test to compare frequencies. For the analysis of the differences between sexes within each age group, we used the Mann–Whitney U test and the Pearson’s chi-squared test, respectively. All the analyses were conducted in Stata (Version 12.0, StataCorp LLC, College Station, TX, USA), with the statistical significance set at *p* < 0.05.

## 3. Results

### 3.1. Demographics

A total of 47,549 individuals of the EpiChron Cohort died at the age of 80 years or older at some point from 1 January 2011 to 31 December 2015; 1255 of them were centenarians. The demographic, clinical, and health services use characteristics of the study population are shown in Table 1 and Table S1. The proportion of women was higher in centenarians (77.7% vs. 50.2% in octogenarians). The average age of centenarians was 101.6 years, and the maximum age was 111 in women and 109 in men. The proportion of people who had lived in an urban area was significantly higher in centenarians, in both sexes. The proportion of elders belonging to the less deprived administrative health areas (i.e., Q1) was higher in centenarians (30.5% vs. 26.1% in octogenarians). Some differences by sex regarding the type and deprivation index of the residential area were observed in people aged 80–99, but not in centenarians (Table 1).

### 3.2. Multimorbidity

Clinical characteristics of the study population regarding the burden and prevalence of chronic diseases are shown in Table 1 and Table 2. We found significantly lower multimorbidity rates in centenarians (78.2% vs. 95.0% in octogenarians). We did not detect differences regarding multimorbidity prevalence between sexes. Moreover, centenarians had a significantly higher proportion of individuals free of chronic conditions (6.0% vs. 1.0%) and lower burden of chronic diseases compared with octogenarians (6.82 vs. 3.76 diagnoses).

Centenarians showed lower prevalence rates of all chronic conditions compared with younger elders, except for chronic ulcer of the skin. Hypertension was the most common chronic condition in the three age groups, although its prevalence decreased with age from 68.0% in octogenarians to 52.6% in centenarians. Moreover, the prevalence of lipid metabolism disorders and diabetes mellitus, the second and third most frequent conditions in octogenarians, substantially decreased with age.

Regarding sex, some cardiovascular risk factors such as hypertension, disorders of lipid metabolism and obesity (but not diabetes) were significantly more prevalent in women than in their male counterparts in all age groups. We observed the same trend for dementia, chronic ulcers of the skin, varicose veins of lower extremities, and osteoporosis. On the other hand, conditions such as chronic obstructive pulmonary disease, gout, low back pain, and renal calculi were significantly more prevalent in men. We also found that prevalence for many other chronic conditions presented significant differences between the two sexes, but these differences were not significant in centenarians (Table 2 and Table S2). Although cardiac arrhythmia, ischemic heart disease, acute myocardial infarction, generalized atherosclerosis, cerebrovascular disease, and peripheral vascular disease were significantly more prevalent in men than in women, these differences in centenarians were not significant. We observed the same for some types of malignant neoplasms, such as colorectal, lung, and high impact neoplasms. On the contrary, depression, anxiety and neurosis, as well as hypothyroidism and asthma, were more prevalent in women but with no significant differences between the two sexes in centenarians.

### 3.3. Polypharmacy

Information regarding drug use is shown in Table 1 and Table 3. The prevalence of polypharmacy was lower in centenarians (50% vs. 77% in octogenarians), and it was higher in women in all age groups, but this difference was significant only in octogenarians. The average of dispensed medications dropped from more than seven drugs in octogenarians to less than five in centenarians. Approximately 7% of centenarians had no chronic medications dispensed, while only 3% of octogenarians were free of chronic drug dispensations. 

We found that potentially inappropriate chronic medications had been dispensed to most of the elders during the 12-month-period before death. The prevalence of patients that had been treated with high anticholinergic risk drugs (Anticholinergic Drug Scale Score ≥3) was approximately 15% in octogenarians and 8% in centenarians. 

Gastroprotective drugs (50%), antithrombotic agents (40%), analgesics and antipyretics (36%), diuretics (33%), anxiolytics (23%), and antidepressants (15%) were the most frequently dispensed chronic drugs in all age groups. The dispensation of antithrombotic agents was substantially lower in centenarians compared with younger elders. Among women, the prevalence of patients with a chronic dispensation of analgesics and antipyretics was significantly lower in centenarians. Lipid modifying agents and blood glucose-lowering drugs, excluding insulin, were two of the ten most dispensed drugs in octogenarians but not in the older adults. 

In the three age groups analyzed, women had a significantly higher prevalence of dispensations of anxiolytics, opioids and calcium; on the other hand, men had higher dispensations of inhalant adrenergic and antigout preparations. The dispensation of high-ceiling diuretics and angiotensin II receptor blockers was higher in women, but this difference was not significant in centenarians. We did the same observation for antipsychotics, hypnotics, and sedatives. We found that dispensation of antithrombotic agents, plain lipid-modifying agents, and vasodilators for cardiac diseases was higher in men, but these differences were not significant in centenarians. The information on differences in the dispensation of the complete list of chronic drugs is presented in Table S3.

### 3.4. Health Services Use

Information on health services used by the study population is shown in Table 1. Primary care was the level of care most frequently visited by the elders, and we found that centenarians used it less. We observed that healthcare services (primary care and specialty/hospital care) were used more frequently by men than women, but these differences were not significant in centenarians. We observed that centenarians less used both primary care services and specialties; also, the number of visits to different specialties was smaller. Whereas 69% of octogenarians visited a specialist, only 29% of centenarians used specialty care, with an average of 2.7 visits during the 12-month-period before death.

## 4. Discussion

This study confirmed that more women than men reached the age of 100, with a female centenarian population approximately 3.5 times higher than the male one. Many authors published similar observations [25,26]. It is relevant to mention that this proportion was not that high among individuals who died at the age of 90–99 years old, and it was almost equal for men and women in octogenarians. 

Results regarding health status and multimorbidity burden in centenarians vary greatly among studies; some centenarians live with high morbidity burden and in poor health, others reach exceptional longevity relatively healthy and functional [3,4,6,12,27]. Different methodological approaches regarding the data sources (e.g., EHRs, surveys, self-reported data) and definitions used (e.g., chronic condition, polypharmacy, chronic medication) could partially explain these discrepancies. In our study, we exhaustively searched for all chronic diseases registered in the EHRs of a real-world cohort, and we found that a centenarian had approximately three chronic conditions less than an individual who died at the age of 80–89 years old. An interesting finding is that preventable risk factors like hypertension, lipid metabolism disorders, and diabetes were the most prevalent chronic conditions among individuals who died between 80–89 years of age (i.e., almost one in three had diabetes, and two in three had hypertension), whereas all cardiovascular risk factors including obesity were significantly less prevalent in centenarians. In centenarians, cardiovascular and cerebrovascular diseases were the most common conditions, although cardiovascular diseases were less prevalent than in younger elders, especially cardiac arrhythmia. The high prevalence of cardiovascular diseases in centenarians is a common finding in the literature [3,4,26,27,28,29,30]. One retrospective study in non-hospitalized centenarians dying of sudden natural death reported that all individuals had at least one disease [31]; aortic dilatation was a constant finding. The same study revealed that 60% of the deceased were described as having been healthy before death, and 58% of them had cardiovascular disease and 23% hypertension.

Based on James F Fries’ theory of the compression of comorbidity [13], Evert J. et al. identified three types of centenarians regarding the age of onset for age-related diseases: survivors (onset before the age of 80), delayers (onset at the age of 80–99), and escapers (without the diagnosis of the ten common age-associated diseases investigated) [4]. Forty-three per cent of the centenarians were delayers, and 19% were escapers, suggesting that most of the centenarians had none of the ten chronic diseases included in the study before the age of 80. They found that the prevalence of hypertension was higher in centenarian women than in men, as our results also suggest, but the mean age of onset was 86 years in men and 77 years in women. In the same study, the mean onset age for heart disease was 90 years in men and 89 years in women. 

Ailshire J.A. et al. reported a significant heterogeneity in the ageing experience of centenarians before reaching 100 years of age; although centenarians were healthier throughout their 80s and 90s compared with their shorter-lived cohort counterparts, over half of them aged with at least one chronic disease [27]. However, since our data did not include the age of onset of the diseases, and we also lacked laboratory information (molecular data), we could not study the evolution of each pathologic condition regarding differences in temporal and biochemical characteristics.

Besides cardiovascular diseases [32,33], we observed that many amongst the most prevalent chronic diseases in centenarians were conditions that have been associated in the medical literature to low-grade elevations in levels of circulating inflammatory mediators, like diabetes [34,35], dementia [36,37,38], and osteoporosis [39]. Low-grade inflammation is one of the key risk factors for mortality [40]. In our study, these diseases were widespread in centenarians, but their prevalence was higher in younger elders. 

Our data revealed that the majority of our elders were poly-medicated with at least one potentially inappropriate medication. Polypharmacy is a common issue in both primary and secondary healthcare, and it is strongly associated with increasing age and multimorbidity [41]. In our study, a centenarian had used approximately 2.5 different drugs less than an individual who died at the age of 80–89 years old and presented a better security profile regarding drugs’ anticholinergic activity. The most common drugs in all age groups were those prescribed for peptic ulcer and gastro-esophageal reflux disease; although less prevalent in centenarians than in younger elders, this was the most dispensed medication. In Sweden, the prevalence of drugs for peptic ulcer and gastro-esophageal reflux disease was much lower, approximately 15% in community-dwelling centenarians and below 20% in institutionalized centenarians [25]. The following most used drug classes in our study were antithrombotic agents and high-ceiling diuretics. Antithrombotic agents were more used in men, whereas high-ceiling diuretics were more common in women, but these differences were not significant in centenarians. The prevalence of high-ceiling diuretics was lower in centenarians than in patients died at the age of 80–89, but in Sweden, this drug class was the most prevalent in centenarians, and it was less common in nonagenarians and octogenarians. In the same study, other types of cardiovascular drugs (like beta-blockers and angiotensin-converting enzyme inhibitors) were less common in centenarians compared with nonagenarians and octogenarians, as we also observed in our results [25].

During their last year of life, centenarians had fewer visits to primary care services (general practitioner or nurse), fewer hospital admissions, shorter length of stay, and fewer visits to a specialist than younger elders. Added to the lower burden of chronic diseases [42], the lower prescription rates in centenarians could explain, at some point, the less intensive use of healthcare services in this age group, as the number of medications is directly related to the risk of potential interactions and adverse side effects [43]. In elders, and especially in centenarians, limited access of a patient to healthcare services due to physical or psychological dependency and to patient’s preferences, with consequent underdiagnosis of his/her pathologic conditions, should be considered when interpreting these results.

The main strength of this work is that this study draws on a population-based cohort representative of the Spanish population, although only the public health system users were analyzed. Moreover, all the clinical variables and those regarding health services use were extracted from patients’ EHRs. In addition, data in the EpiChron Cohort undergo continuous quality control check-ups. Therefore, this information should be more reliable and accurate than if it had been self-reported by patients, potentially subject to recall bias. Moreover, we comprehensively analyzed comorbidity by using an exhaustive list of chronic conditions and not only the most prevalent or severe ones. Regarding drug dispensation data, the information was also highly reliable as it was obtained from pharmacy billing records that represent the medications finally dispensed to the patient. One of the main limitations is that we were not able to collect and analyze specific variables related to longevity that could have been of interest for the study, such as lifestyle habits; functional situation; and biological, educational, and socioeconomic indicators. Moreover, this is a descriptive study of the main clinical characteristics of older people during their last year of life, and no veracious information can be obtained on which factors are specifically associated with a lower or greater likelihood of becoming a centenarian, which would require further study. On the other hand, patients’ clinical history constitutes a primary source of clinical information; however, it is not primarily designed for research purposes and, consequently, could entail some errors during the registration process and potential over- or under-reporting of specific conditions. 

## 5. Conclusions

Although multimorbidity and polypharmacy were the rule in our elders during the year prior to death, the centenarians presented lower morbidity and treatment burden and less use of healthcare services than octogenarians and nonagenarians. This suggests an apparently better health status and leaves the door open to recognizing that this population dies more as a consequence of a natural biological ageing mechanism than of the presence of specific diseases. Sex-differences in the clinical characteristics were more striking in the octogenarian population and tended to become less evident as age increased. Identifying potential factors for exceptional longevity would require further multidisciplinary longitudinal studies that should simultaneously analyze clinical, biochemical, and lifestyle information.

## Figures and Tables

**Table 1:** Demographic, clinical, and health services use characteristics of the study population by age group (dead at the age of 80–89, 90–99, or ≥100 years) and sex (M, men; W, women).

Information	Octogenarians			Nonagenarians			Centenarians				
Demographic	M	W	*p*	M	W	*p*	M	W	*p*	*p*_men_	*p*_women_
Total population (*n*)	14,184	14,280		6282	11,548		280	975			
Age (mean) *	84.6	85.2	**<0.001**	92.7	93.3	**<0.001**	101.7	101.6	0.248		
Residence (urban, %)	48.3	51.7	**<0.001**	49.2	52.5	**<0.001**	58.9	56.8	0.530	**0.001**	**0.006**
Deprivation index (%) ^a^			**<0.001**			**<0.001**			0.317	**<0.001**	**0.002**
Q1	24.4	27.8		27.7	30.1		29.4	30.8			
Q2	23.4	22.8		23.0	22.1		24.0	22.4			
Q3	23.7	22.8		22.9	22.7		25.1	21.1			
Q4	28.5	26.6		26.5	25.1		21.5	25.7			
**Clinical**											
Free of chronic disease (%)	1.0	1.0	0.548	1.8	1.6	0.341	8.1	5.5	0.123	**<0.001**	**<0.001**
Chronic diseases (mean) *	6.76	6.87	**0.008**	5.86	5.63	**<0.001**	3.77	3.75	0.817	**<0.001**	**<0.001**
Multimorbidity ^b^ (%)	94.7	95.3	**0.022**	92.2	91.7	0.239	76.9	78.6	0.573	**<0.001**	**<0.001**
Free of chronic medication (%)	2.8	2.6	0.297	3.2	3.4	0.450	9.2	6.5	0.169	**<0.001**	**<0.001**
Chronic drugs (mean) *	7.32	7.59	**<0.001**	6.71	6.60	0.069	4.89	4.92	0.864	**<0.001**	**<0.001**
Polypharmacy ^c^ (%)	76.1	78.3	**<0.001**	72.0	70.7	0.092	48.2	50.8	0.490	**<0.001**	**<0.001**
With potentially inappropriate medications ^d^ (%)	79.9	84.2	**<0.001**	79.9	82.0	**0.001**	69.3	72.3	0.371	**0.001**	**<0.001**
Potentially inappropriate medications (mean) *	1.59	1.80	**<0.001**	1.61	1.72	**<0.001**	1.28	1.37	0.426	**0.001**	**<0.001**
ADS ^e^ score (%)			**<0.001**			**<0.001**			0.691	**0.001**	**<0.001**
0	40.7	35.4		41.6	37.8		52.8	49.0			
1	29.3	30.3		29.9	31.5		28.4	30.0			
2	15.6	18.1		15.4	17.3		10.1	12.5			
≥3	14.4	16.3		13.1	13.4		8.7	8.6			
ACB ^f^ score (%)			**<0.001**			**<0.001**			0.086	**0.001**	**<0.001**
0	45.4	40.4		44.5	40.7		56.9	50.4			
1	28.0	28.3		27.8	29.3		25.2	30.4			
2	10.0	11.8		11.4	12.6		5.5	9.2			
≥3	16.7	19.6		16.3	17.3		12.4	10.0			
**Health Services Use**											
Users of Primary Care (%)	86.3	86.3	0.984	82.8	84.3	**0.010**	75.0	77.6	0.354	**<0.001**	**<0.001**
Visits to GP ^g^ (mean) *	16.0	15.0	**<0.001**	14.6	13.1	**<0.001**	10.9	11.3	0.750	**<0.001**	**<0.001**
Visits to Nurse (mean) *	14.6	14.5	**<0.001**	12.5	12.6	**0.001**	10.1	13.3	0.140	**<0.001**	**<0.001**
Users of specialties (%)	73.8	65.1	**<0.001**	59.1	46.7	**<0.001**	28.6	28.6	0.527	**<0.001**	**<0.001**
Visits to a specialist (mean) *	8.31	7.03	**<0.001**	4.99	4.29	**<0.001**	2.79	2.77	0.480	**<0.001**	**<0.001**
Different specialties visited (mean) *	3.22	2.84	**<0.001**	2.28	1.97	**<0.001**	1.40	1.49	0.760	**<0.001**	**<0.001**
Users of hospital (%)	39.2	33.9	**<0.001**	30.6	24.8	**<0.001**	15.4	13.1	0.338	**<0.001**	**<0.001**
Hospital admissions (mean) *	1.79	1.67	**<0.001**	1.51	1.47	0.415	1.33	1.34	0.891	**<0.001**	**<0.001**
Length of stay in days (mean) *	18.9	18.2	0.149	15.3	14.8	0.284	10.4	11.8	0.560	**<0.001**	**<0.001**
Users of emergency room (%)	15.4	14.2	**0.004**	14.5	12.8	**0.002**	10.7	8.0	0.153	**0.032**	**<0.001**
Visits to emergency room (mean) *	2.20	2.06	**0.023**	1.93	1.83	0.102	1.90	1.55	0.279	**<0.001**	**<0.001**

* A non-parametric test was used. ^a^ From less (Quartile 1, Q1) to most (Quartile 4, Q4) deprived administrative health areas. ^b^ Defined as the presence of two or more diseases from a list of 114 conditions. ^c^ Defined as five or more drugs dispensed. ^d^ According to the updated Beers criteria. ^e^ Anticholinergic Drug Scale. ^f^ Anticholinergic Cognitive Burden. ^g^ General Practitioner. *p*_men_ and *p*_women_ represent the *p* values of the comparisons among men of different ages and women of different ages. To facilitate the reading of the table, significant *p* values are highlighted in bold, and 95% confidence intervals of the means are not shown (the complete table is provided as Table S1).

**Table 2:** Prevalence of chronic conditions of the study population by age group (dead at the age of 80–89, 90–99, or ≥100 years) and sex (M, men; W, women).

	Octogenarians			Nonagenarians			Centenarians				
Chronic Condition ^a^	M	W	*p*	M	W	*p*	M	W	*p*	*p*_men_	*p*_women_
Hypertension	63.7	72.7	**<0.001**	58.6	68.8	**<0.001**	44.2	55.0	**0.003**	**<0.001**	**<0.001**
Lipid metabolism disorders	31.9	34.9	**<0.001**	19.0	22.1	**<0.001**	5.8	10.0	**0.047**	**<0.001**	**<0.001**
Diabetes	30.6	30.6	0.977	21.0	21.5	0.477	10.0	11.9	0.472	**<0.001**	**<0.001**
Dementia	19.5	28.5	**<0.001**	20.9	29.7	**<0.001**	14.6	22.4	**0.008**	**0.007**	**<0.001**
Cardiac arrhythmia	27.2	23.9	**<0.001**	24.8	20.0	**<0.001**	13.9	10.9	0.226	**<0.001**	**<0.001**
Cerebrovascular disease	23.1	22.0	**0.021**	25.8	24.1	**0.012**	21.9	20.5	0.673	**<0.001**	**<0.001**
Cataract, aphakia	23.9	24.3	0.414	22.4	18.7	**<0.001**	13.1	12.2	0.777	**<0.001**	**<0.001**
Congestive heart failure	20.3	22.4	**<0.001**	23.4	23.8	0.626	18.9	21.1	0.475	**<0.001**	**0.014**
Degenerative joint disease	17.5	27.0	**<0.001**	17.2	23.3	**<0.001**	11.2	14.8	0.167	**0.026**	**<0.001**
Chronic ulcer of the skin	16.2	21.3	**<0.001**	18.3	26.4	**<0.001**	24.6	31.3	**0.047**	**<0.001**	**<0.001**
Depression	14.4	27.2	**<0.001**	12.7	19.9	**<0.001**	6.5	7.4	0.720	**<0.001**	**<0.001**
Varicose veins of lower extremities	11.4	27.0	**<0.001**	11.6	22.9	**<0.001**	6.5	15.0	**0.001**	**0.041**	**<0.001**
Emphysema, chronic bronchitis, COPD ^b^	28.0	10.4	**<0.001**	24.1	9.0	**<0.001**	14.6	5.6	**<0.001**	**<0.001**	**<0.001**
Iron deficiency, other deficiency anemias	16.6	16.8	0.666	16.2	14.7	**0.008**	10.8	9.4	0.580	**0.034**	**<0.001**
Prostatic hypertrophy	32.1	0.0	**<0.001**	31.0	0.0	**<0.001**	23.9	0.0	**<0.001**	**0.007**	**0.033**
Osteoporosis	4.8	23.9	**<0.001**	4.1	17.6	**<0.001**	1.5	7.9	**<0.001**	**0.008**	**<0.001**
IHD ^c^ (excl. AMI ^d^)	15.7	10.1	**<0.001**	13.4	9.6	**<0.001**	8.5	7.0	0.508	**<0.001**	**0.007**
Glaucoma	11.2	11.0	0.551	12.9	11.2	**0.001**	8.1	9.1	0.715	**0.001**	0.143
Surgical aftercare	13.4	12.4	**0.013**	9.2	7.8	**0.002**	3.5	2.8	0.727	**<0.001**	**<0.001**
Other neurologic disorders	11.3	12.5	**0.002**	10.0	9.2	0.068	4.6	4.3	0.967	**<0.001**	**<0.001**
Deafness, hearing loss	10.2	10.4	0.577	12.2	10.5	**0.001**	7.7	9.5	0.444	**<0.001**	0.626
Other respiratory disorders	12.6	9.9	**<0.001**	11.2	8.8	**<0.001**	6.9	4.5	0.162	**0.001**	**<0.001**
Dermatitis and eczema	11.1	9.5	**<0.001**	10.3	8.7	**<0.001**	8.1	5.6	0.186	0.084	**<0.001**
Obesity	9.7	14.0	**<0.001**	4.9	7.0	**<0.001**	0.8	3.3	**0.044**	**<0.001**	**<0.001**
Other cardiovascular disorder	10.5	10.7	0.584	7.6	7.4	0.785	1.9	3.5	0.294	**<0.001**	**<0.001**
Cardiac valve disorders	8.6	9.6	**0.002**	5.1	5.0	0.736	1.2	1.8	0.592	**<0.001**	**<0.001**
Other endocrine disorders	5.5	10.7	**<0.001**	3.9	7.4	**<0.001**	2.3	4.2	0.218	**<0.001**	**<0.001**
Chronic renal failure	9.6	6.5	**<0.001**	8.4	5.0	**<0.001**	3.9	3.3	0.839	**<0.001**	**<0.001**
Hypothyroidism	3.5	10.3	**<0.001**	2.8	6.6	**<0.001**	2.3	2.9	0.758	**0.016**	**<0.001**
Acute myocardial infarction	9.1	4.3	**<0.001**	7.7	3.9	**<0.001**	3.9	2.9	0.571	**<0.001**	**0.050**
Thrombophlebitis	5.0	6.9	**<0.001**	4.7	6.6	**<0.001**	2.7	5.2	0.130	0.197	0.125
Visual impairment	6.1	5.6	0.066	6.6	5.7	**0.019**	6.5	3.9	0.096	0.417	0.073
High impact MN ^e^	7.9	6.2	**<0.001**	3.8	2.4	**<0.001**	1.2	1.0	0.732	**<0.001**	**<0.001**
Parkinson’s disease	6.7	5.8	**0.001**	4.6	3.8	**0.012**	1.5	0.8	0.270	**<0.001**	**<0.001**
Other hematologic disorders	6.3	5.8	0.056	4.7	3.3	**<0.001**	3.1	1.5	0.116	**<0.001**	**<0.001**
Diverticular disease of colon	5.5	5.7	0.580	4.2	4.2	0.856	2.3	2.1	0.989	**<0.001**	**<0.001**
Sleep disorders of nonorganic origin	4.9	4.4	0.059	5.6	4.7	**0.010**	5.8	4.9	0.661	0.158	0.661
Asthma	3.1	7.1	**<0.001**	2.7	5.3	**<0.001**	1.5	3.0	0.278	0.163	**<0.001**
Gout	8.5	2.2	**<0.001**	7.2	2.0	**<0.001**	6.2	1.9	**0.001**	**0.004**	0.367
Low back pain	5.6	4.6	**<0.001**	4.2	2.7	**<0.001**	3.9	1.2	**0.013**	**<0.001**	**<0.001**
MN of skin	5.0	3.0	**<0.001**	6.3	3.9	**<0.001**	5.0	3.3	0.286	**0.001**	**<0.001**
MN, prostate	8.7	0.0	**<0.001**	7.8	0.0	**<0.001**	4.6	0.0	**<0.001**	**0.008**	0.149
MN, colorectal	5.0	3.0	**<0.001**	3.1	2.0	**<0.001**	1.9	1.6	0.785	**<0.001**	**<0.001**
Anxiety, neuroses	2.3	4.8	**<0.001**	2.3	3.3	**<0.001**	1.2	2.1	0.443	0.481	**<0.001**
Gastroesophageal reflux	2.8	3.2	0.108	2.7	2.3	0.132	0.8	1.5	0.545	0.110	**<0.001**
Peripheral neuropathy, neuritis	2.6	3.0	0.059	2.0	1.9	0.492	0.4	1.3	0.319	**0.005**	**<0.001**
Irritable bowel syndrome	2.0	2.9	**<0.001**	2.2	2.1	0.960	0.8	1.3	0.746	0.269	**<0.001**
Generalized atherosclerosis	3.3	2.0	**<0.001**	2.3	1.5	**<0.001**	0.4	0.7	1.000	**<0.001**	**<0.001**
Seizure disorder	2.3	2.9	**0.003**	1.6	1.8	0.536	2.3	1.1	0.134	**0.007**	**<0.001**
MN, bladder	4.6	0.9	**<0.001**	3.2	0.6	**<0.001**	1.9	0.8	0.150	**<0.001**	**0.025**
MN, breast	0.0	3.9	**<0.001**	0.0	2.4	**<0.001**	0.0	1.0	0.219	1.000	**<0.001**
Pulmonary embolism	1.6	2.2	**0.001**	1.8	1.5	0.219	0.4	0.8	1.000	0.211	**<0.001**
Chronic respiratory failure	2.6	1.5	**<0.001**	1.3	1.2	0.859	0.8	0.2	0.210	**<0.001**	**0.001**
Disorders of immune system	2.2	1.9	0.088	1.5	1.2	0.138	1.5	0.7	0.239	**0.007**	**<0.001**
Peripheral vascular disease	3.0	1.2	**<0.001**	1.7	0.9	**<0.001**	1.5	0.4	0.075	**<0.001**	**0.012**
Utero-vaginal prolapse	0.0	3.5	**<0.001**	0.0	2.5	**<0.001**	0.0	1.3	0.080	0.284	**<0.001**
Paralytic syndromes, other	2.2	1.9	0.115	1.4	1.2	0.217	0.8	0.4	0.617	**<0.001**	**<0.001**
Schizophrenia and affective psychosis	1.4	2.3	**<0.001**	1.0	1.3	0.112	0.8	0.7	0.689	0.085	**<0.001**
Kyphoscoliosis	0.9	2.3	**<0.001**	0.6	2.0	**<0.001**	0.0	0.8	0.358	0.065	**0.005**
Psoriasis	2.3	1.3	**<0.001**	1.6	0.9	**<0.001**	0.8	0.2	0.210	**0.003**	**<0.001**
Psychosocial disorders of childhood	1.0	1.7	**<0.001**	1.5	1.9	**0.046**	1.2	2.3	0.382	**0.013**	0.201
Diabetic retinopathy	1.7	2.1	**0.012**	0.8	0.9	0.476	0.0	0.8	0.358	**<0.001**	**<0.001**
Renal disorders, other	2.3	1.3	**<0.001**	1.4	0.8	**0.001**	0.4	0.5	1.000	**<0.001**	**0.001**
MN, lung	3.3	0.7	**<0.001**	1.1	0.3	**<0.001**	0.0	0.1	1.000	**<0.001**	**<0.001**
Substance use	2.5	0.7	**<0.001**	1.3	0.5	**<0.001**	0.4	0.8	1.000	**<0.001**	0.221
Disease of hair/hair follicles	0.5	2.1	**<0.001**	0.7	1.4	**<0.001**	0.4	0.5	1.000	0.652	**<0.001**
Developmental disorder	1.2	1.1	0.281	1.1	1.1	1.000	0.4	0.5	1.000	0.445	0.290
Chronic liver disease	1.9	1.1	**<0.001**	0.8	0.3	**<0.001**	0.4	0.0	0.219	**<0.001**	**<0.001**
Renal calculi	1.6	0.8	**<0.001**	1.0	0.5	**<0.001**	1.5	0.1	**0.009**	**0.003**	**0.001**

^a^ From a list of 114 chronic conditions listed in descending order of total prevalence (only those with prevalence equal to or greater than 1% are represented). ^b^ Chronic obstructive pulmonary disease. ^c^ Ischemic heart disease. ^d^ Acute myocardial infarction. ^e^ Malignant neoplasms. *p*_men_ and *p*_women_ represent the *p* values of the comparisons among men of different ages and women of different ages. Significant *p* values are highlighted in bold.

**Table 3:** Prevalence of chronic medications dispensed in the study population by age group (dead at the age of 80–89, 90–99, or ≥100 years) and sex (M, men; W, women).

	Octogenarians			Nonagenarians			Centenarians				
Chronic Medication ^a^	M	W	*p*	M	W	*p*	M	W	*p*	*p*_men_	*p*_women_
Drugs for peptic ulcer and GORD ^b^	63.5	66.8	**<0.001**	61.5	62.5	0.196	50.5	50.2	1.000	**<0.001**	**<0.001**
Antithrombotic agents	57.8	50.9	**<0.001**	56.6	49.2	**<0.001**	41.7	39.1	0.519	**<0.001**	**<0.001**
High-ceiling diuretics	35.1	38.3	**<0.001**	39.0	41.3	**0.004**	27.5	34.5	0.062	**<0.001**	**<0.001**
Other analgesics and antipyretics	32.2	41.7	**<0.001**	32.3	41.5	**<0.001**	32.6	36.8	0.276	0.984	**0.024**
Antidepressants	20.8	35.2	**<0.001**	21.4	28.5	**<0.001**	12.4	15.5	0.290	**0.005**	**<0.001**
Anxiolytics	17.9	30.4	**<0.001**	18.8	28.5	**<0.001**	14.2	24.9	**0.001**	0.109	**<0.001**
Lipid modifying agents, plain	29.1	26.2	**<0.001**	14.6	12.7	**0.001**	6.0	3.6	0.158	**<0.001**	**<0.001**
ACE ^c^ inhibitors, plain	17.8	16.8	**0.043**	17.0	17.4	0.588	7.3	16.7	**0.001**	**<0.001**	0.507
Blood glucose lowering drugs, excl. insulins	19.5	19.0	0.278	12.9	12.6	0.639	4.6	6.4	0.411	**<0.001**	**<0.001**
Antipsychotics	13.9	16.9	**<0.001**	15.9	18.9	**<0.001**	12.8	14.9	0.503	**0.001**	**<0.001**
Iron preparations	14.2	15.2	**0.020**	16.1	15.3	0.203	13.3	11.0	0.412	**0.004**	**0.004**
Beta blocking agents	16.4	17.4	**0.030**	11.7	11.6	0.798	5.1	5.8	0.812	**<0.001**	**<0.001**
Drugs used in benign prostatic hypertrophy	31.1	0.0	**0.000**	32.4	0.0	**<0.001**	28.0	0.0	**<0.001**	0.117	**0.028**
Hypnotics and sedatives	10.9	15.2	**<0.001**	12.8	16.8	**<0.001**	10.6	15.3	0.095	**0.001**	**0.006**
Non-steroid anti-inflammatory and antirheumatic products	14.4	17.2	**<0.001**	10.4	11.7	**0.014**	8.7	6.4	0.285	**<0.001**	**<0.001**
ARBs ^d^, plain	14.0	16.1	**<0.001**	10.9	13.2	**<0.001**	6.0	7.5	0.537	**<0.001**	**<0.001**
Adrenergics, inhalants	18.3	9.0	**<0.001**	14.3	7.4	**<0.001**	6.9	3.4	**0.038**	**<0.001**	**<0.001**
Selective Ca channel blockers with vascular effect	12.3	12.2	0.853	11.6	11.5	0.830	12.4	9.8	0.321	0.363	**0.036**
Opioids	9.8	15.7	**<0.001**	7.7	11.7	**<0.001**	2.8	6.6	**0.045**	**<0.001**	**<0.001**
ARBs, combinations	11.3	14.6	**<0.001**	7.9	11.1	**<0.001**	5.1	6.9	0.418	**<0.001**	**<0.001**
Antiglaucoma preparations and miotics	11.2	10.5	0.088	12.8	10.3	**<0.001**	7.8	7.3	0.935	**0.001**	**0.015**
Vasodilators for cardiac diseases	12.3	8.6	**<0.001**	13.3	10.4	**<0.001**	7.8	10.4	0.308	**0.020**	**<0.001**
Anti-dementia drugs	10.2	14.3	**<0.001**	6.7	8.4	**<0.001**	2.3	2.1	0.794	**<0.001**	**<0.001**
Other drugs for OAD ^e^, inhalants	16.2	6.6	**<0.001**	12.3	5.8	**<0.001**	6.4	4.3	0.254	**<0.001**	**0.002**
Capillary stabilizing agents	5.2	12.7	**<0.001**	5.7	11.4	**<0.001**	4.4	11.1	0.050	0.503	0.067
Topical products for joint and muscular pain	7.3	11.0	**<0.001**	7.7	9.0	**0.039**	6.2	9.1	0.425	0.655	**0.001**
Drugs for constipation	7.6	8.6	**0.002**	9.5	9.7	0.629	10.1	9.1	0.737	**<0.001**	**0.016**
Antiepileptics	8.5	10.4	**<0.001**	6.0	6.6	0.133	1.8	2.8	0.570	**<0.001**	**<0.001**
Cardiac glycosides	7.1	8.7	**<0.001**	8.1	9.1	**0.031**	7.8	6.4	0.548	0.059	**0.028**
Expectorants, excl. cough suppressants	10.4	6.1	**<0.001**	10.2	6.5	**<0.001**	10.6	7.2	0.140	0.956	0.281
Potassium-sparing agents	8.9	8.7	0.594	6.5	6.6	0.819	6.0	3.7	0.188	**<0.001**	**<0.001**
Psychostimulants, ADHD ^f^ agents, nootropics	7.1	7.2	0.816	7.7	8.0	0.483	9.6	6.5	0.146	0.131	**0.023**
Antigout preparations	11.7	5.2	**<0.001**	9.4	3.7	**<0.001**	5.1	1.8	**0.014**	**<0.001**	**<0.001**
Vitamin B12 and folic acid	7.1	7.7	0.093	7.7	6.9	**0.046**	5.1	5.5	0.921	0.158	**0.009**
Calcium	3.9	11.7	**<0.001**	2.3	7.4	**<0.001**	0.5	3.2	**0.045**	**<0.001**	**<0.001**
Insulins and analogues	7.4	9.4	**<0.001**	3.9	4.8	**0.006**	1.4	1.7	1.000	**<0.001**	**<0.001**
Selective calcium channel blockers with cardiac effects	7.1	7.0	0.826	5.6	6.2	0.135	2.8	2.7	1.000	**<0.001**	**<0.001**

^a^ According to their Anatomical Therapeutic Chemical classification system code at the third level and listed in descending order of total prevalence (only those with prevalence equal to or greater than 5% are represented). ^b^ Gastro-esophageal reflux disease. ^c^ Angiotensin-converting enzyme. ^d^ Angiotensin II receptor blockers. ^e^ Obstructive airway diseases. ^f^ Attention deficit hyperactivity disorder. p_men_ and p_women_ represent the *p* values of the comparisons among men of different ages and women of different ages. Significant *p* values are highlighted in bold.

## Supplementary Materials

Supplementary Materials can be found at https://www.mdpi.com/2077-0383/9/5/1563/s1. Table S1: Demographic, clinical, and health services use characteristics of the study population by age group and sex. Table S2: Prevalence of all chronic conditions of the study population by age group and sex. Table S3: Prevalence of all chronic medications dispensed in the study population by age group and sex.
